# Point Sensor Networks Struggle to Detect and Quantify Short Controlled Releases at Oil and Gas Sites

**DOI:** 10.3390/s24082419

**Published:** 2024-04-10

**Authors:** Rachel Elizabeth Day, Ethan Emerson, Clay Bell, Daniel Zimmerle

**Affiliations:** 1 Department of Systems Engineering, Colorado State University, Fort Collins, CO 80523, USA; 2 Energy Institute, Colorado State University, Fort Collins, CO 80524, USA; 3 BPX Energy, Denver, CO 80202, USA

**Keywords:** methane, emissions abatement, continuous monitoring, emissions quantification, oil and gas, greenhouse gas (GHG)

## Abstract

This study evaluated multiple commercially available continuous monitoring (CM) point sensor network (PSN) solutions under single-blind controlled release testing conducted at operational upstream and midstream oil and natural gas (O&G) sites. During releases, PSNs reported site-level emission rate estimates of 0 kg/h between 38 and 86% of the time. When non-zero site-level emission rate estimates were provided, no linear correlation between the release rate and the reported emission rate estimate was observed. The average, aggregated across all PSN solutions during releases, shows 5% of the mixing ratio readings at downwind sensors were greater than the site’s baseline plus two standard deviations. Four of seven total PSN solutions tested during this field campaign provided site-level emission rate estimates with the site average relative error ranging from −100% to 24% for solution D, −100% to −43% for solution E, −25% for solution F (solution F was only at one site), and −99% to 430% for solution G, with an overall average of −29% across all sites and solutions. Of all the individual site-level emission rate estimates, only 11% were within ±2.5 kg/h of the study team’s best estimate of site-level emissions at the time of the releases.

## 1. Introduction

Anthropogenic emissions are the leading cause of increased atmospheric greenhouse gas (GHG) concentrations in the last 150 years [1]. Atmospheric carbon dioxide (CO_2_) accounts for 79% of human caused GHGs, but methane (CH_4_) has a global warming potential that is roughly 86 times higher than CO_2_ over a 20-year period [2]. The short atmospheric lifetime of CH_4_ (≈12 years) and high warming potential means that a reduction in CH_4_ emissions would have a near-term effect on the radiative balance of the atmosphere and efforts to mitigate climate change [1,3,4]. The 2022 Inflation Reduction Act (IRA) [5] included the Methane Emissions Reduction Program, notably a waste emissions charge for sites emitting over 25,000 metric tons of CO_2_ equivalent (CO_2_e). Recent proposed changes to Subpart W for petroleum and natural gas systems in the Greenhouse Gas Reporting Program (GHGRP) contain improvements to the existing calculation methodologies to supplement calculated CH_4_ emission factors with direct measurements [6]. Further, in December 2023, the US Environmental Protection Agency (EPA) published the final OOOOb New Source Performance Standards (NSPS) and OOOOc Emission Guidelines (EG) for oil and gas sites which includes standards to allow operators to use continuous monitoring solutions as an alternative means of emission detection [7]. In order for measurements to improve the accuracy of emission inventories, or for continuous emissions monitoring systems (CEMs) to provide a robust equivalent alternative to prescribed leak detection methods, it is imperative that measurements from these systems are repeatable, accurate, and unbiased.

A typical North American onshore production site includes surface equipment to perform the first separation of production fluids into condensate (oil), natural gas, and produced water. Natural gas and condensate are transported through pipelines to larger, more complex compressor stations and/or gas processing plants where the gas is further refined to marketable natural gas and natural gas liquids.

Operational emissions on production sites occur during routine processing and maintenance, including activities such as flaring, venting, compressing, dehydrating, and heating. Unintentional emission sources include fugitives (e.g., threaded connections, flanges, valve packing seals, and other component leaks) and process malfunctions (e.g., unlit flares, or stuck liquid dump valves on separation vessels, which result in excess venting at liquid storage tanks). While fugitive component leaks have been the subject of traditional leak detection and repair (LDAR) practices, they often exhibit relatively low emission rates [8,9]. More recently, process malfunctions have been identified as high emission rate sources potentially responsible for the discrepancies between bottom-up inventories and top-down measurement studies, and which may be both readily detected and abated [10,11,12,13,14,15,16].

Until recently, LDAR techniques for detecting fugitive methane emissions were performed manually by operators who maintain the sites or third party contractors hired to perform onsite inspections. Traditional LDAR techniques involved Method 21, optical gas imaging (OGI), or audio, visual, and olfactory (AVO)-type inspections, all of which are manual and time intensive processes to inspect each equipment unit and component [17]. Next-generation leak detection and quantification (LDAQ) solutions attempt to provide a less time-intensive methodology and are generally divided into two types, based upon the deployment and supervision of the solution [18,19,20,21,22,23,24]. Survey solutions detect and quantify emissions during ‘snapshots’ in time, and CEMs monitor emissions ‘continuously’. Survey solutions are typically handheld or mobile systems, and collect data on emissions for short periods, seconds to hours, to detect and quantify emissions. CEMs consist of sensors, analytics, and a dashboard to convey results to end users autonomously [5].

In contrast to traditional voluntary and regulatory LDAR methods, next generation LDAQ solutions (including survey and CEM solutions) use gas sensors and/or wind measurements coupled with algorithms to detect emissions and provide some combination of emission event detection, localization, and/or per-emitter or per-site emission rate estimates using proprietary algorithms [18,25]. Hybridized approaches leveraging alternative detection systems to find high emitting sources more quickly to achieve equivalent or more emission reductions, and thereby relaxing the frequency of traditional LDAR required to detect component leaks, have also been proposed [6].

A subset of CEMs, point sensor networks (PSNs), use stationary point sensors to provide a continuous (e.g., 1 hz) measurement of the methane mixing ratio (ppm) at the location of each sensor [26]. Commercially available PSNs utilize a variety of sensor types including optical, capacitance-based, calorimetric, resonant, acoustic-based, pyroelectric, semiconducting metal oxide (SOM), and electrochemical sensors [26]. The cost, sensitivity, gas selectivity, power requirement, and other specifications of each sensor type influence the selection by commercial product developers.

Safety restrictions at operational O&G sites typically require solutions to be installed at the perimeter or along the fenceline of sites. Some solutions have sought certifications allowing them to be installed in areas classified as potentially explosive environments, enabling them to be located within the fenceline and closer to equipment. Most PSNs also install an anemometer at each site to measure the local wind speed and direction. Figure S1 in the Supplementary Material shows an example of point sensors that were implemented on one of the field campaign’s sites. If accurate, PSNs could provide O&G operators with an efficient and continuous way of monitoring operational and unintentional emissions.

This study is one phase of a larger program to develop methodologies to test the performance of emission detection and quantification solutions, named Advancing the Development of Emission Detection (ADED), funded by the U.S. Department of Energy (DOE) with contributions from partner operators and solution developers. ADED includes elements of LDAQ solution testing in both controlled conditions and field deployments. ADED developed controlled release (CR) test protocols for both CEMs and survey methods, which have been implemented at Colorado State University’s Methane Emissions Technology Evaluation Center (METEC) [27]. These protocols include instructions on single-blind testing and performance metrics, including probability-of-detection (POD), quantification accuracy, and localization precision, on a per-emitter basis [28].

The CRs performed at METEC followed the CEM testing protocol while releasing natural gas from a confined and controlled tubing network through surface-mounted retired equipment donated from O&G operators [28]. The equipment at METEC is representative of upstream and midstream onshore O&G sites in North America; however, there are no ‘operational emissions’ (pneumatic venting, incomplete combustion, packing vents, etc.) occurring at METEC as none of the equipment was operating or attached to non-controlled natural gas lines. Performance evaluation and accuracy of 11 CEMs, six of which were PSNs, was conducted at METEC in 2022 and 2023 following the consensus CEM protocol established [27,29]. The 2022 and 2023 METEC studies involved CRs of measured and recorded natural gas flows from locations simulating emissions on the modeled O&G site equipment [27]. CRs were regulated to provide release duration and flows based on site constraints and detection limits defined by CEMs solution developers. During CR testing at METEC, CEM solution developers provided detection reports for CRs and results from both years show reasonable performance for detection (90% POD from 0.006 to 7.1 kg/h) at a site where no operational emissions occur, but high uncertainty (underestimation and overestimation by factors up to >15 and 97, respectively) [29] for emissions rate estimates.

During the field campaign for this study, single-blind controlled release experiments were conducted at active oil and gas locations, including upstream production and midstream gathering sites, to evaluate the field performance of commercially available continuous monitoring, emission detection, and measurement solutions. In this study, we will refer to a controlled release conducted at operational oil and gas sites as a challenge release (ChR), while a release performed at METEC will be referenced as a CR. The term ChR is used as a reminder that the flow of the release was controlled, metered, and recorded; however, coincident operational emissions at the active O&G sites are unknown. A ChR, therefore, reflects a minimum emission rate for the site at the time of the release, or a delta from a non-zero baseline expected in the site-level estimates from a PSN during the release.

## 2. Materials and Methods

This study considers ChR testing undertaken at operational O&G sites in the United States (US) to evaluate and compare PSNs’ field performance at real O&G sites with performance during METEC testing to identify if results of METEC testing are indicative of the solutions’ ability to identify unintentional emission sources in field conditions [27,29]. Note, specifics of the METEC testing does not replicate routine operational emission sources, such as exhaust emissions or venting from pneumatic controllers seen at sites [27]. Three fundamental differences between the field campaign and METEC were (1) the solution deployments, (2) the operational nature of the active O&G sites where the field campaign took place, and (3) the format of data provided for evaluation of detection and quantification performance.

The field campaign was performed by the ADED research team with the participation of O&G partner operators. These operators provided access to host sites, deployed solutions and provided access to the solution data, and supplied natural gas for the ChRs (see ‘Challenge Release Equipment’ below). Operator personnel were on-site with the field teams continually for all of the ChRs.

ChRs took place at seven O&G production sites and four gathering stations in the Upper Green River (Wyoming), Marcellus (Pennsylvania), Utica (Ohio), and Permian Basins (Texas) in 2022 and 2023. The field campaign included three total deployments, numbered 1–3 and the solutions that participated in the field campaign are labeled A–G. Production sites included general extraction equipment, such as wellheads, separators, combustion exhaust sources, dehydrators, flares, etc., and were smaller than the gathering stations, which included several compressors, slug catchers (a type of liquid separator on inlet gas lines), pig launchers and receivers, vapor recovery units, tanks, and miscellaneous other equipment. Some of the gathering stations included additional processing equipment, such as stabilizers and de-ethanization towers. On average, production sites included in the study were 3.5 acres and the gathering stations were 10.4 acres (Supplementary Material Table S1). By comparison the area used for METEC studies was smaller, at 1.5 acres. Supplementary Material Table S1 details site type, equipment, size, and provides data information for all deployments of the field campaign. Operators, solutions, and sites are coded with letters to maintain anonymity.

### 2.1. Solution Deployment

A total of six solution developers participated in the field campaign, and there were seven total PSN solutions tested. One of the developers tested two different solutions during the field campaign. Each operator selected PSN solutions to deploy at their sites using their discretion for the testing period. Therefore, not all PSN solutions were deployed at each site. The following solutions deployed at least once during the field campaign: Baker–Hughes’ Lumen Terrain, Project Canary’s Canary X, Project Canary’s SCS Nubo Sphere, Earthview’s BluBird, Qube’s Axon, Sensirion Nubo Sphere, and ChampionX’s SOOFIE. Operators installed solutions at their sites prior to the field campaign. Solutions were installed by the operators following guidance on sensor placement from the solutions themselves; however, in most cases only general guidance (for example “install at corners of site”) was provided and little guidance was given to select specific locations. In most cases, solutions were installed around the perimeter of the site often coinciding with the site property boundary or surrounding fence line. No solutions were designated for installation or operation in a classified area (e.g., a Class I Div I environment). Sensor positions during challenge releases were logged by the study team and are shown overlaid on satellite imagery of each site in Supplementary Material Section S2.1. The study team for this work was independent of the solution developers and interested in the performance of the sensor systems’ ability to detect, localize, and quantify methane emissions at active oil and gas facilities. The study team coordinated with the oil and gas facility operators who, in turn, coordinated the installation, commissioning, and operation of PSN solutions at their respective facilities. The study team did not participate or supervise the installation of monitoring systems at locations.

At METEC, solution developers deployed their own sensors using their desired installation strategy, provided it met the safety requirements of METEC. In both the field campaign and at METEC, the solutions deployed one or more anemometers to measure the wind speed and direction.

### 2.2. Challenge Release Equipment

For the field campaign, a mobile release rig was used for ChRs at the host sites, allowing gas to be released at metered rates from locations where methane emissions may occur. Supplementary Material Figure S5 provides an example of a release location at a host site. Supply for the release rig was provided by a field tap into the operator’s sales or conditioned fuel gas line. Supplementary Material Figure S6 provides an example of where the release rig pulled gas at a host site. Since the supply gas was typically from a location downstream of liquids separation or other processing, gas used for ChRs may have had a higher methane fraction than other potential unintentional emission sources at the host sites.

The release rate was controlled by adjusting the flow path to different sized precision orifice flow restrictors, and could be fine-tuned by adjusting an upstream regulator. The regulator could be bypassed to achieve higher release rates, or when operating from a low-pressure gas supply system, such as a conditioned fuel gas system post regulation (Supplementary Material Figures S7 and S8). ChRs were metered by a Fox FT2 mass flow meter calibrated for the range of controlled release experiments. Timestamped release rate data were logged at 1 hz by an on board microcomputer, and the location of each release point was manually recorded by the study team. The release rig was manually controlled to provide a continuous emission at a constant emission rate for the duration of a release and only a single ChR was conducted at a time. Multiple ChRs were sometimes conducted in succession from the same ChR location using different release rates for different durations.

### 2.3. Challenge Releases

**Figure 1:**
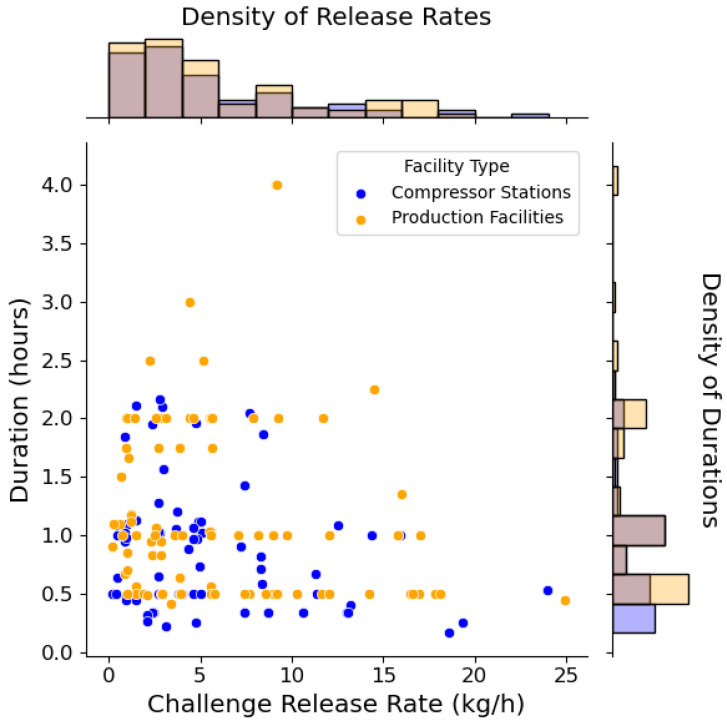
Duration versus release rate of 90 ChRs conducted at production sites and 75 ChRs conducted at compressor stations. The field campaign consisted of ChR rates ranging from 0.2 to 24.1 kg/h that lasted for 10 to 240 min from representative fugitive leak or vented locations using the transportable controlled release rig. Supplementary Material Figures S9 and S10 show separate histograms for duration of ChRs and ChR rates.

The ChRs during the field campaign served to simulate an additional, unintentional emission with a known release rate to the baseline operational emissions from the site. Most of the operational emission sources at these sites were continuous: compressor exhaust and packing seals, unburnt methane from catadyne heaters on meter runs, and reboilers for combination units (dehydration and separation). Only a few intermittent sources were present (gas operated pneumatics, and in some cases maintenance work caused short blowdowns or vents). ChR rates were originally chosen based on typical fugitive component emission rates (0–2 kg/h) [8,30,31,32] and discussions with the operator; however, after PSN systems initially struggled to detect these lower ChR rates, the planned release rates were modified to include higher emission rates in an attempt to improve the learnings from the study (Figure 1).

Across all host sites, 165 ChRs were performed in total. All releases were conducted during weekday operations (M–F) between 8 a.m. and 5 p.m. with the supervision/participation of operator personnel. Duration ranged from 10 to 240 min (average 68 min) with rates between 0.2 and 24.1 kg/h (average 5.2 kg/h). The portable release rig was setup to a field tap, a release location was decided with the operator, and the emission point was temporarily installed at the selected location.

CRs at METEC were similar to the ChRs in the field campaign, with two key differences. First, at METEC, there were no un-metered emissions from on-site operations. Therefore, solutions could identify any release as an emission without having to establish a non-zero baseline of emissions from the site. Second, during METEC testing, the study team monitored solution reports and manipulated the emission rate so that each solution achieved a near 100% detection probability at some release rate (typically large), and a near 0% detection probability at another release rate (typically small). Moving release rates in this way effectively ‘mapped out’ the POD curve for most solutions. This approach requires 300–400 experiments for each solution. In contrast, far fewer releases were possible for each solution in the field campaign, and the overall poor performance, even at release rates approaching the upper limit of the release system and far greater than typical fugitive component leaks, made it impractical to map the curve.

### 2.4. PSN Solution Data

During the field campaign, the solutions did not provide detection reports using the same email-based reporting method as required during METEC testing [28]. Instead, the study team was granted access to the solution’s “dashboard”, a graphical user interface provided to operators to receive alerts, interact with data, investigate or acknowledge detections, and export raw or processed emission data from the solutions. Exportable data varied between solutions; Supplementary Material Table S1 provides information on each solution’s data provided. Solutions (D), (E), (F), and (G) provide averaged site-level emission rate estimates in increments of 10, 1, 15, and 15 min, respectively. Most provided time series of methane or the total hydrocarbon gas mixing ratio from each point sensor. Some also provided site-level emission rate estimates. Site-level emission rate estimates also varied, including probability of release location tables based on equipment groupings, or alert tables with coordinates of estimated release locations. Data frequency also varied across solutions and across data types for a given solution. For example, data products from one solution included methane mixing ratios at 1 Hz, site-level emission rate estimates at 5 min intervals, and a most probable source location(s) at a daily resolution.

Independent measurement of all the operational emission sources at a given site was not conducted due to challenges coordinating a time-coincident independent measurement, limitations of direct measurement techniques, and other complicating factors. Instead, the field team used data from the continuous monitors when the field team was not running ChRs (weeks preceding and after); these data are identified as non-release (NR) data. NR data were utilized to compare site-level emission rate estimates with/without active ChRs. Note that the field campaign did not measure a background site methane emission baseline from leak screening (e.g., optical gas imaging [33,34]) or measurement by a downwind method (such as OTM33 [35,36]). The mean of NR site-level emission rate estimates from each solution was used to represent what the solution would report in the absence of a ChR at a given site, hereafter referred to as ‘baseline (BL)’. These values can be compared to site-level emission rate estimates during ChRs for the same site–solution pair to determine if the presence of a ChR impacted the site-level estimate. The amount of available NR data varied for site–solution pairs, and ranged from one to six weeks.

The original intent of the field campaign was to utilize the same metrics as METEC CR testing, specifically, POD, quantification accuracy, and localization accuracy [28]. Since solutions did not provide defined detection reports that could be used for this purpose, the field team needed to interpret the dashboards’ raw data to determine if there was a sufficient change in emissions that the presence of excess emissions at the site could be reasonably identified. To avoid subjective bias, this was completed by defining thresholds for what change in emissions constituted a detection. Further, these thresholds needed to be applicable to all solutions. The analysis used thresholds that could be applied to exportable data from the solutions’ dashboards, specifically:

1. Mixing ratio data taken from the solutions’ sensors
2. Site-level methane emission rate estimates, hereafter ‘emission estimates’.

Several analyses were performed, and the thresholds specific to each analysis are provided along with the results below.

### 2.5. Challenge Release Detection Classification

Solutions can be configured to alert at operator-defined emission thresholds and/or durations. Since operators did not have much time to configure solutions prior to the testing, these automated alerts were not enabled or leveraged in the detection classification. Due to limitations in the reported data, the study team could not identify true positive detections in a robust and meaningful way for solutions A, B, and C. The majority of emissions estimates from solutions D, E and G, were 0 kg/h. For these solutions, ‘any non-zero emission estimate overlapping in time with a challenge release was classified as a true positive (TP)_POD_ detection.

This TP_POD_ definition is conservative and accepts any non-zero estimate during the ChR as a TP_POD_, regardless of attribution, indicating the detection was of our release, not some other activity or operational emission at the site. Solution F did not report any 0 kg/h emission estimates, and a TP_POD_ detection was defined as any emission estimate above the site BL; that is, if any site-level emission estimate greater than the BL was reported during the ChR, the ChR was designated as a TP_POD_. For any solution, if a ChR was not classified as a TP_POD_ detection following the logic above, then it was classified as a false negative (FN)_POD_ detection. An FN_POD_ is defined as a non-detect, meaning the challenge release was not identified by the solution. POD curves were then derived from TP_POD_ and FN_POD_ data using the regression methodology, following METEC testing [29]. False positives and true negatives could not be attributed during these studies, because the field team was unable to rule out the presence of all fugitive or vented emissions from operational activities at the site.

Therefore, a classification matrix and the non-parametric $\chi^{2}$ statistical test of independence was used to assess whether a statistical difference may exist in a solution’s data between the reported site-level emission rate estimates when ChRs were occurring versus when they were not [37]. As solutions A, B, and C did not report emission rate estimates, this analysis was only conducted for solutions D, E, F, and G. A contingency table, or classification matrix, was formed for the $\chi^{2}$ test with counts for rows of emission estimates and columns of release types. The associations between rows and columns of the classification matrix are found through Equation (1) and hypothesis testing, where for this study, the null hypothesis is that there is no association between emission estimates during ChRs and estimates when we were not releasing.

(1) $$\chi^{2}=\sum \frac{{\left(o_{x,y}-e_{x,y}\right)}^{2}}{e_{x,y}}$$

where o_x,y_ is the observed count in row x and column y, and e_x,y_ is the expected count. If $\chi^{2}\leq 0.05$ (p≤0.05), then the null hypothesis is accepted. Different from the POD definition for detection, the classification matrix used reported NR emission estimates to identify a TP_E_ detection or a FN_E_ non-detection. Note that the $\chi^{2}$ statistic does not identify a relationship; a significant result (p≤0.05) indicates only that a relationship cannot be ruled out. Classification was applied to any site-level emission estimate, E_i,j_, for solution i, at site j, such that:

(2) $$TP_{E}\leftarrow E_{i,j}\geq {\bar{E}}_{\mathrm{NR},i,j}+\sigma_{E_{\mathrm{NR},i,j}}$$

(3) $$FN_{E}\leftarrow E_{i,j}<{\bar{E}}_{\mathrm{NR},i,j}+\sigma_{E_{\mathrm{NR},i,j}}$$

where $\bar{E}$_NRi,j_ is the mean of all NR reports by solution i at site j, and $\sigma_{E_{\mathrm{NR},i,j}}$ is the standard deviation of all NR reports by solution i at site j.

For the mixing ratio analysis, we first identify downwind sensors as any sensor which is within ±45° of directly downwind from the ChR location (Supplementary Material Figure S11). All other sensors are classified as ‘not downwind.’ Solution F did not provide maxing ratio values, so they were not included in this analysis. A TP_X_ and FN_X_ sensor response, X_i,j_ is defined as any reported mixing ratio by a downwind sensor where:

(4) $$TP_{X}\leftarrow X_{i,j}\geq {\bar{X}}_{\mathrm{NR},i,j}+2\sigma_{X_{\mathrm{NR},i,j}}$$

(5) $$FN_{X}\leftarrow X_{i,j}<{\bar{X}}_{\mathrm{NR},i,j}+2\sigma_{X_{\mathrm{NR},i,j}}$$

Higher percentages of TP_X_ responses at the downwind sensors compared with the upwind sensors could indicate that the sensors are picking up a response when directly downwind of a ChR.

### 2.6. Quantification Analysis

To assess quantification performance, we compare the solutions’ emission estimates in NR conditions to estimates when ChRs were occurring. This analysis assumes the site-level emission estimate (zero or non-zero) during NR periods represents the baseline operational emissions at the site and any ChR represents an incremental emission source which the solution should detect. The relative error, ϵ, for solution i during a ChR at site j was defined as:

(6) $$\begin{array}{c} \epsilon_{i,j}=\frac{\sum E_{i,j}-\sum \left(SOE\right)}{\sum (SOE)} \end{array}$$

where the study onsite estimate (SOE) is the sum of the ChR rate, c_j_, and the BL, b_i,j_, and E_i,j_ is the site-level emission estimate provided by solution i at site j. If E_i,j_ accurately reflected the additional emissions from the ChR, E_i,j_ = SOE and the relative error is zero. This method is analogous to the use case, where operators wish to be notified of unexpected fugitive emissions; that is, the solution must establish a baseline emission rate from the site, and then accurately assess the presence of incremental emissions. This analysis is also analogous to the “action-levels” defined in OOOOb NSPS, where a deviation of 1.2 kg/h (for wellhead only sites) or 1.6 kg/h (for other affected facilities) in the rolling 90-day average over a site-specific baseline requires a follow-up action. [7] Additionally, the percent of emission estimates E_i,j_ that were within ±2.5 kg/h of the SOE were found for each site and each solution.

Given the observed POD performance, a classification matrix approach was also conducted to determine if a relationship exists between the quantification estimates with/without ChRs. The analysis used a 3×3 classification matrix with experiments classified along one axis, and the emission estimates classified along the other. Experiments were classified into three groups: “Not releasing” when no ChR is active, “ChR ≤ BL” when a ChR is lower than the solution’s BL estimate of the site, and “ChR > BL” when a ChR is larger than the solution’s BL estimate of the site. Site-level emission estimates were classified as “Zero Estimate” when E_i,j_ = 0, as “Within 3x” when $\frac{{SOE}_{i,j}}{3}\leq E_{i,j}\leq 3\cdot \left({SOE}_{i,j}\right)$, or as “Outside 3x” when $E_{i,j}>3\cdot \left({SOE}_{i,j}\right)\;\mathrm{or}\;E_{i,j}<\frac{{SOE}_{i,j}}{3}$.

(7) $$Zero\;Estimate\leftarrow E_{i,j}=0$$

(8) $$Within\;3x\leftarrow \frac{SOE_{i,j}}{3}\leq E_{i,j}\leq 3\cdot SOE_{i,j}$$

(9) $$Outside\;3x\leftarrow \left[\begin{array}{c} E_{i,j}>3\cdot SOE_{i,j}\\ \mathrm{or}\\ E_{i,j}<\frac{SOE_{i,j}}{3} \end{array}\right]$$

## 3. Results

**Figure 2:**
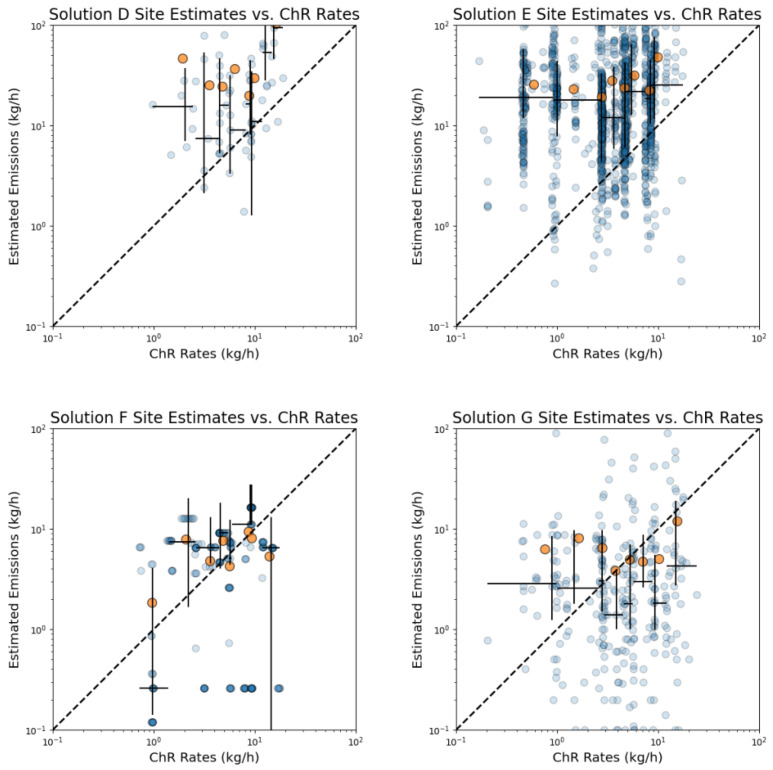
Solutions’ site-level estimates from all sites temporally aligned with ChRs. Individual estimates are shown as blue circles. Data were separated into bins with equal points and plotted as orange dots to indicate the average estimated emission rate. Horizontal whiskers indicate the bin width, vertical whiskers indicate the 25th and 75th percentiles for estimated emission rates and the intersection is the median. Estimates of 0 kg/h are not included in this log-log plot.

Four of the seven solutions provided site-level emission rate estimates, with solutions frequently reporting 0 kg/h (38%-G, 62%-E, and 86%-D). Excluding 0 kg/h estimates, no clear relationship between challenge release rates and solutions’ site-level emission rate estimates were observed during the field campaign across all sites (Figure 2). Solutions D and E show high bias for all ChR rates, while solution F and G show high bias at low ChRs rates and low bias during the higher ChR rates. A solution that is sensitive to the ChRs amongst the site’s background emissions would have shown a linear relationship above the 1:1 line and indicates an insensitivity to the tested conditions.

In a non-parametric Spearman correlation analysis, emission estimates were compared with various parameters, including mixing ratio readings, wind speed and direction, ChR rate, and sensor density. The analysis revealed the strongest correlation with mixing ratio readings, registering a Spearman’s ρ value of 0.34. This outcome aligns with expectations, considering that the proprietary algorithms used to generate emission estimates incorporate these mixing ratio readings as a fundamental component of their calculations. Wind speed exhibited a low correlation of 0.17. In contrast, ChR rate, wind direction (expressed as wind angle), and sensor density demonstrated negligible correlations with Spearman’s ρ values of −0.027, 0.006, and −0.002, respectively.

The notably low correlation observed between the ChR rate and emission estimates underscores potential inaccuracies within either the sensor measurements or the algorithms employed for emission estimation. This is further evidenced by the absence of a linear correlation as depicted in Figure 2. Such findings highlight the critical need for scrutinizing the methodologies applied in deriving emission estimates, particularly the reliability of the sensors and the robustness of the algorithms utilized.

There was a substantial spread observed between controlled releases and solutions’ estimates during controlled testing at METEC, indicating a wide uncertainty in these solutions’ estimates for any given release [29]. These uncertainties are exacerbated in the field campaign by the operational nature of a site where during any given challenge release, the site-level emission rate estimates from any one solution often span many orders of magnitude. Supplementary Material Figures S12–S22 show solution site-level estimates versus SOEs for each site and solution pair.

### 3.1. Probability of Detection

**Figure 3:**
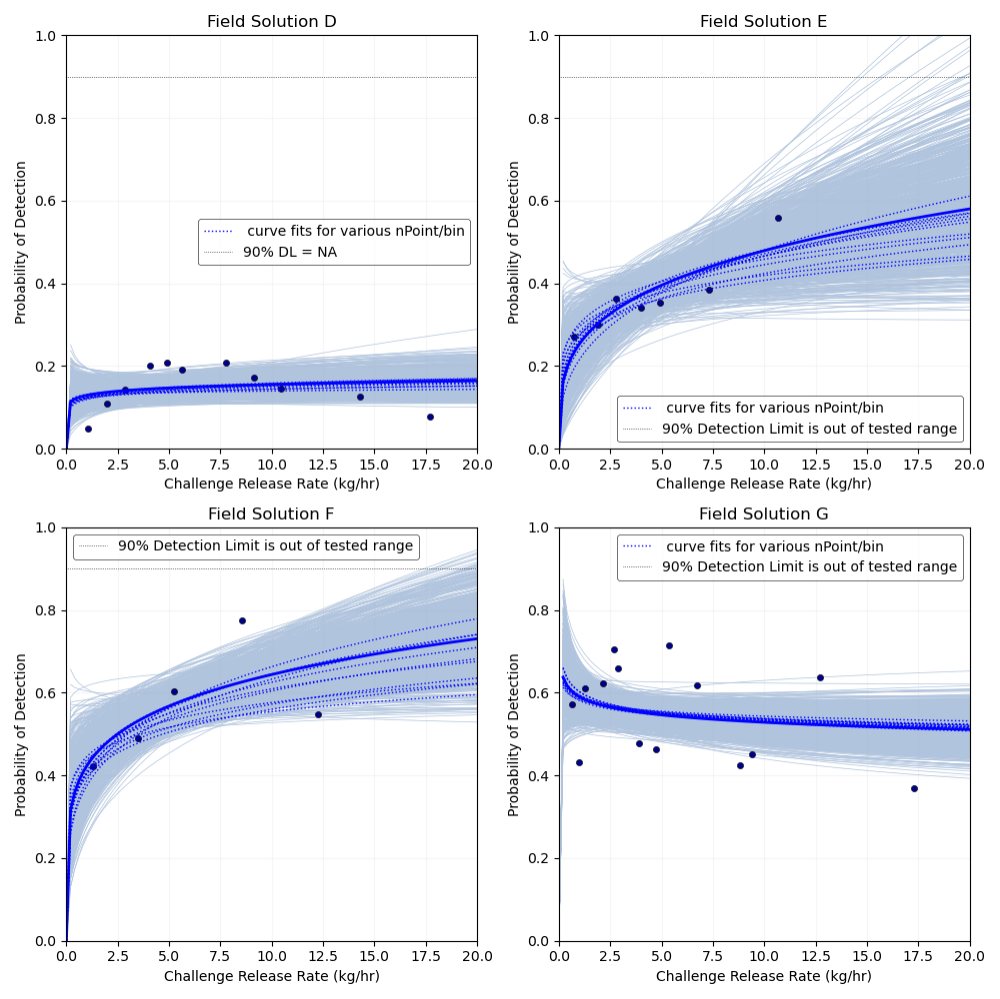
Probability of detection as a power curve function of site rate estimates during ChRs for the four solutions with emission rate estimates during all deployments. The ChR rates in the x-axis are split into equal sized bins, with the dot marker showing the average ChR rate per bin. The definition of detection, or a TP_POD_ reading, for the field campaign includes any estimate above 0 kg/h for solutions D, E, and G. As solution F does not have any 0 kg/h estimates, the definition of detection is any estimate above 2.23 kg/h, the BL site rate estimate, see Section 2.

None of the solutions achieved a 90% POD across the range of ChRs conducted, as shown in Figure 3. Implementing the METEC POD framework to the field campaign results in substantially reduced performance at operational sites when comparing the same solutions’ METEC POD curves. None of the solutions demonstrated POD results similar to that in METEC testing, as shown in the logistic regression POD curve in Supplementary Material Figure S23. This suggests the test and analysis methods utilized for METEC CR testing provided little insight into actual field performance. One variance in the test method between METEC and the field campaign that may have affected the results was the number of sensors per area. Each solution that participated in METEC testing deployed more sensors per acre at METEC than at any location in the field campaign (Supplementary Material Figure S24). This leads to increased “blind-spots” in the field deployments where a ChR may disperse between sensors and not transect any sensor location downwind for the duration of the experiment. While this implies a lower POD, and our field results confirm, it is important to recognize the ChR in this study were relatively short in duration (0–4 h) and a CM solution performance may improve given longer opportunities to detect where the wind may have increased directional variability. However, controlled releases at METEC were generally of similar duration, with the large majority lasting between 0 and 4 h.

The non-parametric classification shows 85% of the emission estimates made during ChRs and 94% of estimates during NR periods were below the detection threshold. To determine if the change in emission estimates between periods with ChRs and without ChRs have a chance of significance, the results of the $\chi^{2}$ test from each site–solution combination are summarized in Table 1. Note that the $\chi^{2}$ test does not confirm a relationship between the solution response and the presence/absence of a ChR; significance only indicates that such a relationship cannot be ruled out. The results indicate that no difference is observed between periods with/without ChRs in 11 of the 19 site–solution combinations. Of the 19 combinations, all solutions indicated the possibility of a detection relationship at least once, including solution F which tested in only one combination.

### 3.2. Mixing Ratio Results

The poor relationship between ChRs and detections may be driven by multiple factors which may also vary between solutions. However, a successful detection for any solution would require two sequential events to be true: (a) the solution’s sensor must respond to the ChR with increased readings, and (b) the solution’s algorithms must identify a detection by successfully analyzing the sensor data. We analyze (a) by reviewing the time series of the mixing ratio data as per Equations (4) and (5). Unlike the emission rate-based classification, this was possible for all solutions tested, except for solution F.

From site–solution combinations that provided reviewable mixing ratio data, sensors downwind averaged 5% of readings indicating enhancements greater than 2$\sigma_{X_{\mathrm{NR},i,j}}$, while sensors upwind averaged 1% of readings indicating enhancements. Since sensors were ≈100 m from the ChR emission sources, see Supplementary Material Table S2, the low 5% enhancement rate observed during ChRs is unsurprising, given the instability of transport in near-field dispersion. These data indicate the presence of a signal at the sensors, and, therefore, the presence of information which could potentially identify controlled releases.

However, the signal is both weak and noisy, likely indicating that post-processing algorithms require improvement to extract detections from the signal. Increasing the number of sensors deployed at a facility and decreasing the average distance from source to sensor may result in improvement by increasing the odds that at least one sensor is downwind of any given source and increasing the signal-to-noise ratio, respectively.

**Figure 4:**
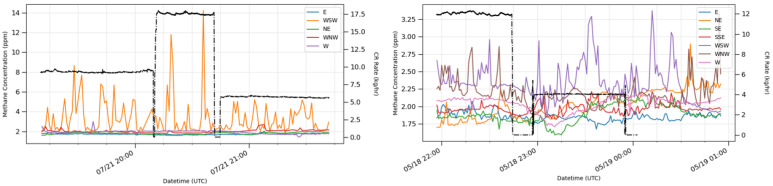
Solution C’s mixing ratio estimates in comparison with the ChR rates at Site 1. The black dashed line shows the ChR rate and the colored lines are mixing ratio measurements from the point sensors at the site. (**Left**) panel illustrates a period with wind from the NW and the closest downwind WSW sensor measuring peak mixing ratios that increase and decrease with the ChR rate. Other sensors which are not downwind of the ChR show little response. (**Right**) plot shows the same solution at Site 1, with the wind direction moving through a section with no sensors. Mixing ratio enhancements are, therefore, not present in any sensor, and variability in the sensor mixing ratio is random or associated with routine operational emission sources at the site.

Figure 4 provides an example of the enhancement analysis, showing sensor activity with respect to the ChR rates. Under ideal sensor positioning and wind directions, a ChR from a location occurring directly upwind of a sensor node of the PSN shows a mixing ratio enhancement where the peak mixing ratios trend with different ChR release rates (Figure 4, left panel); changes in the mean mixing ratios are less clear. During varied wind directions, the enhancements do not trend with the ChR release rate, and a period with no ChR shows readings similar to periods with releases (Figure 4, right panel).

Figure 4 shows one example; other site–solution combinations displayed similar behavior with varying degrees of clarity. These qualitative results suggest that algorithms may need to consider multiple wind transport parameters to know when mixing ratio enhancements are likely to occur, over what upwind angle, at what intensity, and there may be a need to modify both detection and quantification algorithms to match the meteorological conditions. For conditions outside of operable parameters, observations are unlikely to be indicative of emissions, and may need to be discarded. This would result in fewer emission reports of higher accuracy than the data provided by algorithms at the time of testing.

### 3.3. Site Rate Quantification Results

While the study design was primarily intended to evaluate detection and alerting of unintentional emission sources using ChRs, many solutions are now attempting to provide site-level, time-resolved emission rate estimates. In this mode, detection of any given emitter is of lower priority, and accurate estimates of site-level emissions over extended periods are higher priority. Recent regulatory changes including the Inflation Reduction Act (IRA), proposed amendments to the US GHGRP, and the EPA’s Final Methane Rule raise the priority for this mode [7]. With the new waste emission charge starting in 2024 at $900/tonnes above defined emission intensities, the solutions’ emission estimate accuracy is of importance to O&G companies and regulatory authorities. Additionally, the Final Methane Rule allows CEM solutions to be implemented by operators as an alternative means for fugitive emission detection using site-level emission rate-based action limits. The rule specifies action levels for sites with major production and processing equipment, centralized facilities, and compressor stations as a deviation of 1.6 kg/h in a 90-day rolling average and a deviation of 21 kg/h in a 7-day rolling average above a site-specific baseline.

Table 2 shows the solutions’ estimates averaged at each site during the field campaign and extrapolated to an annual estimate by assuming the ChR continued at the average emission rate for a full year (8760 h). All solutions underestimate the magnitude of additional emissions from the ChRs relative to the solution’s BL. This analysis highlights the implications of inaccurate site-level emission estimates resulting from the application of proprietary inversion models used by PSNs at the time of testing, where the assessed waste emission charges may be substantially biased (in this case low) relative to the true site annualized emissions. Note that this analysis only considers the difference between a site-level emission rate estimate and the BL during a ChR compared to the magnitude of the ChR and does not consider the accuracy of the solution’s BL itself or if the waste emission charge would be applicable based on the methane intensity threshold. Therefore, the study does not conclude that CM emission estimates would result in reduced charges for operators relative to actual emissions, but instead may only conclude that the accuracy of emission estimates from PSNs is not sufficient to base a waste emission charge on. BL emissions assessed by different solutions at each site varied significantly (see Supplementary Material Table S3). Though this study can not assess the accuracy of any one solution, the high variability in the baseline emission estimates across solutions indicates that annualized estimates developed by integrating site-level emission estimates from PSNs versus time are unlikely to provide an accurate estimate of the true annual emissions.

Also as a part of the EPA’s Final Methane Rule, if a certified third party (remote measurement systems that do not rely on access to facilities, e.g., satellite or aerial measurements) detects an emission of 100 kg/h or greater of methane, it will be considered a super-emitter event and the O&G operator will need to take action to address the event [7]. During the times of ChRs in the field campaign, solutions D, E, and G reported emissions greater than or equal to 100 kg/h 3, 46, and 1 times, respectively, even though all ChRs were below 25 kg/h (25% of the EPA’s Super-Emitter Program (SEP) threshold).

Histograms presented in Supplementary Material Figure S25 depict the individual site-level emission rate estimates of the solutions, revealing a prevalence of estimates clustered around or near 0 kg/h at all sites. Substantially higher site-level emission rate estimates are observed at a much lower frequency, particularly in the cases of D, E, and G. This indicates that solutions are missing site emissions. Even estimates of 0 kg/h during NR times are likely inaccurate, due to the presence of operational emissions, particularly at compressor stations where non-zero exhaust emissions from compressors and packing seals are present as well as from heaters and combusters for dehydration systems.

Supplementary Material Figure S26 shows that the average site-level emission rate estimates during ChRs are higher than during NR periods (except solution E). This is in line with expectations and may indicate solutions are working to some degree; however, (a) the variability in emission estimates during any given ChR is large ranging from below the ChR release rate to much higher than the ChR release rate plus the BL, and (b) the TP/FN classification and detection analysis was conservative/forgiving and still indicates poor detection. Table 3 shows the mean relative error for nearly all solutions at nearly all facilities is negative, indicating emission estimates during ChRs were consistently biased low, i.e., a smaller incremental increase above BL was observed during a ChR than the release rate of the ChR.

In Table 4, we present the percentages of non-zero emission estimates falling within a range of ±2.5 kg/h of the SOE. Notably, any 0 kg/h site-level emission rate estimate was considered not within this range, reflecting the expectation that site-level emission rate estimates should not be 0 kg/h during ChR activities. For instance, if a ChR of 0.5 kg/h occurred alongside a baseline of 0.5 kg/h, totaling 1 kg/h of SOE, an emission estimate of 0 kg/h would technically be in range but is excluded from consideration in our analysis. The infrequent alignment of solution estimates within the bounding range and frequent reports of no emissions suggests underlying issues with their estimation accuracy. Note, the band of ±2.5 kg/h is greater than the action level defined in the EPA OOOOb NSPS, indicating that solutions may not currently be capable of providing data with high enough precision to make the rule effective.

Lacking a clear proportional relationship between ChR emission rates and reported emissions, we utilized a classification matrix approach to determine if any relationship *could* exist. From the $\chi^{2}$ tests performed on the quantification matrices, 18 of the 19 site–solution pairs showed that a statistically significant relationship could not be ruled out.

This indicates that the difference between emission estimates when ChRs were occurring and when ChRs were not occurring may not be random, even though little correlation was seen between the deviation from the BL in the reported site-level emission rate estimates and the emission rate of the ChR. With a factor of 3, the limits for the classification matrix provided a wide range for the estimates to fall within, but Table 5 shows only a small amount of site–solution pairs within those limits.

## 4. Discussion

Recent regulatory and voluntary emissions reporting changes will place additional reliance on the detection and measurement of emissions at sites for reporting purposes. To trust any measurement method for this purpose, the performance of the method needs to be understood in two areas.

First, numerous studies have indicated that a small number of large emitters contribute disproportionately to total emissions from O&G sites. A key selling point of CEM is rapid detection of large emitters, shortening the time to detect and mitigate, thus reducing total emissions. Therefore, detection performance is a key input to CEM mitigation performance. This study shows that the field campaign POD is significantly lower than the POD in controlled test conditions at METEC and indicates that controlled testing did not reflect field conditions accurately. Therefore, new methods are needed to translate controlled testing performance into field conditions.

However, field campaigns are unlikely to provide the type of rigorous testing available in controlled testing at a test center. Controlled testing still remains essential for characterizing solution performance. A 12-week test period at METEC covers more than 400 CR experiments, per solution, operating 24 h per day, 7 days per week. In contrast, eight weeks of field deployment in this study enabled only 165 ChR experiments to be conducted, and it was infeasible for all solutions to be installed at all sites for these experiments. This resulted in a small number of experiments, relative to METEC testing, for any single solution. Given this constraint, this study indicates that controlled testing must be improved to better reflect the field conditions.

When the analysis controls for wind conditions and times when emissions are directly upwind of a sensor, the mixing ratio readings when ChRs are active differ from times when ChRs are not active, indicating that a signal exists using current sensor technology. This suggests that point sensors may be sufficient to detect emissions at field sites, but current algorithms seem unable to reliably extract accurate emission rate estimates from the sensor readings. Additional investments in analytics are likely required, although improvements in sensing technologies may also be necessary.

Second, ignoring whether individual incremental emitters (i.e., the ChRs) were detected, there is an interest in using CEM to regularly report the emission rates from sites. To be used in this mode, the total emissions observed by the CEM over an extended period must reasonably represent the total emissions at the site. While the results from the ChRs performed in this study represent a short experimental duration, the results strongly suggest that using CEMs to estimate long-term emissions is inaccurate. In this study, results from the ChRs indicate that most solutions, at most sites, do not accurately report the incremental emissions, represented by ChRs ranging from 0.2–24.1 kg/h. Given that many emitters in field conditions are intermittent, and the sizes utilized here are representative of those emitters, the results suggest long-term reporting will not correctly report the emissions from sites. However, the statistical analysis does not conclude a relationship does not exist between reported emission rates and ChRs. These results suggest that a signal exists, but the current algorithms and deployment methodologies may not be sufficiently advanced to accurately estimate emissions in field conditions, and that further development of CEM analytics is required for this application.

## Figures and Tables

**Table 1:** Results from the emission rate-based detection classification matrix. Note that ‘No’ indicates that the data are random and ‘Yes’ indicates that a statistical significant relationship cannot be ruled out.

Potential Detection					
Site No.	Site Type	D	E	F	G
Site 1	Production	No	No	Yes	No
Site 2	Production				Yes
Site 3	Production				No
Site 4	Production				No
Site 5	Production				Yes
Site 6	Compressor		Yes		Yes
Site 7	Gas plant		Yes		
Site 8	Compressor		Yes		No
Site 9	Compressor	No			No
Site 10	Production	Yes			No
Site 11	Production	No			No

**Table 2:** Annualized emission estimates compared to annualized ChRs. The difference in waste emission charge assumes $900/tonne that will be implemented in the U.S. Inflation Reduction Act. Note that the difference reflects the solution’s inability to measure the difference in site-level emissions resulting from (ChRs), and does not imply their baseline (BL) is accurate, which may result in waste charges being biased low or high overall.

Total Field Campaign Estimate Averages and New Waste Emission Charge					
Solution	Total Average Estimates (kg)	Total ChRs (kg)	Total BLs (kg)	Total SOEs (kg)	Waste Charge Difference Annually ($)
D	640	650	355	1005	$−151,000
E	1500	545	2770	3315	$−712,000
F	375	360	135	495	$−16,000
G	580	835	670	1505	$−285,000

**Table 3:** Relative error between the individual emission estimates and the SOE. Only periods during ChRs are included in the figure. No NR periods (ChR = 0) are included. The percentage is the average relative error for individual site-level emission estimates for each solution.

Emission Estimate Relative Error					
	Site Type	D	E	F	G
Site 1	Production	−32%	−43%	−25%	−47%
Site 2	Production				−41%
Site 3	Production				−54%
Site 4	Production				−52%
Site 5	Production				430%
Site 6	Compressor		−100%		−64%
Site 7	Gas plant		−88%		
Site 8	Compressor		−46%		−72%
Site 9	Compressor	−56%			−10%
Site 10	Production	24%			−99%
Site 11	Production	−100%			−84%
Mean Error		**−35%**	**−70%**	**−25%**	**−51%**

**Table 4:** Percent of emission estimates within ±2.5 kg/h of ChRs and BLs.

Emission Estimates within ±2.5 kg/h					
	Site Type	D	E	F	G
Site 1	Production	2%	1%	36%	7%
Site 2	Production				41%
Site 3	Production				0.2%
Site 4	Production				39%
Site 5	Production				36%
Site 6	Compressor		1%		27%
Site 7	Gas plant		1%		
Site 8	Compressor		6%		25%
Site 9	Compressor	6%			5%
Site 10	Production	0%			0%
Site 11	Production	0%			8%

**Table 5:** Percentages of site quantification estimates with limits of 3 times the expected site emissions. Only showing percentages of estimates made when the ChR was higher than the BL. A dash specifies sites that the study team was not able to release a ChR above the BL.

Quantification Estimates within Limits					
	Site Type	D	E	F	G
Site 1	Production	12%	-	75%	17%
Site 2	Production				40%
Site 3	Production				44%
Site 4	Production				38%
Site 5	Production				35%
Site 6	Compressor		1%		1%
Site 7	Gas plant		-		
Site 8	Compressor		-		1%
Site 9	Compressor	17%			24%
Site 10	Production	0%			0%
Site 11	Production	0%			2%

## Data Availability

The data that support the findings of this study are available from the corresponding author upon reasonable request.

## Supplementary Materials

The following supporting information can be downloaded at: https://www.mdpi.com/article/10.3390/s24082419/s1, Figure S1: Field Point Sensors, Figure S2: Aerial image of sites 1–3, Figure S3: Aerial image of sites 4–7, Figure S4: Aerial image of sites 8–11, Figure S5: Challenge release location, Figure S6: Gas field tap, Figure S7: Mobile release rig, Figure S8: Release rig flow diagram, Figure S9: Challenge release durations, Figure S10: Challenge release rates, Figure S11: Downwind mixing ratio area, Figure S12: Binned estimates Site 1, Figure S13: Binned estimates Site 2, Figure S14: Binned estimates Site 3, Figure S15: Binned estimates Site 4, Figure S16: Binned estimates Site 5, Figure S17: Binned estimates Site 6, Figure S18: Binned estimates Site 7, Figure S19: Binned estimates Site 8, Figure S20: Binned estimates Site 9, Figure S21: Binned estimates Site 10, Figure S22: Binned estimates Site 11, Figure S23: POD logistic regression curve, Figure S24: Sensor per area, Figure S25: Challenge release emission estimate histograms, Figure S26: ChR and NR emission estimate averages; Table S1: Site details, Table S2: Sensor distances, Table S3: Site baselines.
