# Supplementary material for: Point Sensor Networks Struggle to Detect and Quantify Short Controlled Releases at Oil and Gas Sites
Source: Sensors (Basel). 2024 Apr 10;24(8):2419. doi: 10.3390/s24082419 (PMC11054334; doi:10.3390/s24082419)
Supplement: Supplementary file 1 [file sensors-24-02419-s001.zip › sensors-2905870-supplementary.pdf]

Supplementary Information

Point Sensor Networks Struggle to Detect and Quantify Short Controlled Releases at Oil and Gas Sites

April 9, 2024

Contents

S-1 Introduction **S1**

S-2 Materials and Methods **S2**

S-2.1 Solution Deployment S3
S-2.2 Challenge Release Equipment S4
S-2.3 Challenge Release Detection Classification S7

S-3 Results and Discussion **S8**

S-3.1 Probability of Detection S14
S-3.2 Mixing Ratio Results S16
S-3.3 Site Rate Quantification Results S16

List of Figures

S-1 Field point sensors S1
S-2 Aerial image of sites 1 - 3 . . . . S3
S-3 Aerial image of sites 4 - 7 . . . . S3
S-4 Aerial image of sites 8 - 11 . . . . S3
S-5 Challenge release location S4
S-6 Gas field tap S4
S-7 Mobile release rig S5
S-8 Release rig flow diagram S5
S-9 Challenge release durations S6
S-10 Challenge release rates S6
S-11 Downwind mixing ratio area S7
S-12 Binned estimates Site 1 . . . . S8
S-13 Binned estimates Site 2 . . . . S9
S-14 Binned estimates Site 3 . . . . S9
S-15 Binned estimates Site 4 . . . . S10
S-16 Binned estimates Site 5 . . . . S10
S-17 Binned estimates Site 6 . . . . S11
S-18 Binned estimates Site 7 . . . . S11
S-19 Binned estimates Site 8 . . . . S12
S-20 Binned estimates Site 9 . . . . S12
S-21 Binned estimates Site 10 . . . . S13
S-22 Binned estimates Site 11 . . . . S13
S-23 POD logistic regression curve S14
S-24 Sensor per area S15

S-25 Challenge release emission estimate histograms S17
S-26 ChR and NR emission estimate averages S17

List of Tables

S-1 Site details S2
S-2 Sensor Distances S16
S-3 Site baselines S16

S-1 Introduction

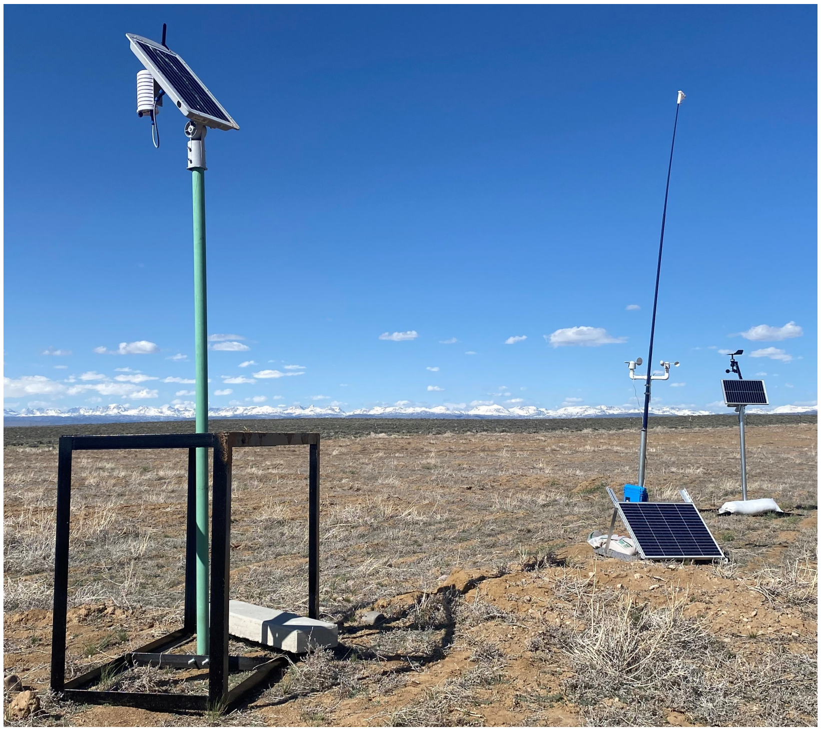

Figure S-1: Image of point sensors implemented on a site during the field campaign.

S-2 Materials and Methods

Table S-1: Review of the field trial's deployments includes information on each site's size, basin, site type, exportable emission data, and site equipment.

Deployment	Basin	Operator	Site Number	Site Size (acres)	Site Type	Solution at Facility	Mixing Ratio Data	Site-Level Emission Estimate Data	Site Equipment
**1**	Upper Green River	1	1	9.5	Production Site	A	✓		Wellheads, line heaters, separators, dehydrators, combustors
						C	✓		
						D	✓	✓	
						E	✓	✓	
						F		✓	
						G	✓	✓	
**2**	Marcellus	2	2	2.5	Production Site	G	✓	✓	Wellhead, sand separator, gas production unit, sales meter, produced water tank
			3	2.5	Production Site		✓	✓	Wellhead, sand separator, gas production unit, sales meter, produced water tank
			4	2.5	Production Site		✓	✓	Wellheads, gas production units, sales meter, pig launcher
			5	2.5	Production Site		✓	✓	Wellheads, gas production units, sales meter, produced water tank, flat top tanks
	Utica	3	6	3.5	Compressor Station	E	✓	✓	Pig receivers and launchers, slug catcher, compressor, vapor recovery unit, bullet tank, flare, natural gas condensate tank, triethylene glycol regenerators, discharge meter
						G	✓	✓	
			7	25	Cryogenic Gas Plant	E	✓	✓	Pig receivers and launchers, slug catcher, cryogenic compressors, hot oil heaters, condensate water tanks, condensate stabilization heaters, produced water tanks, propane chillers
			8	6	Compressor Station	E	✓	✓	Pig receivers and launchers, slug catcher, compressors, inlet scrubber, sales meters, filter seperator, bullet tanks, vapor recovery units condensate pumps, triethylene glycol regenerators, produced water tank, slop oil tank, blowdown stack
						G	✓	✓	
**3**	Permian	4	9	7	Compressor Station	C	✓		Flare, seperators, scrubbers, oil and water tanks, vapor recovery towers, compressors, dehydrators, combustors, scrubber seperators
						B	✓		
						D	✓	✓	
						G	✓	✓	
			10	3.5	Production Site	C	✓		Flares, heater treaters, seperators, gas scrubbers, oil and water tanks, vapor revocery towers
						B	✓		
						D	✓	✓	
						G	✓	✓	
			11	2	Production Site	C	✓		Flares, heater treaters, seperators, gas scrubbers, oil and water tanks, vapor revocery towers
						B	✓		
						D	✓	✓	
						G	✓	✓	

S-2.1 Solution Deployment

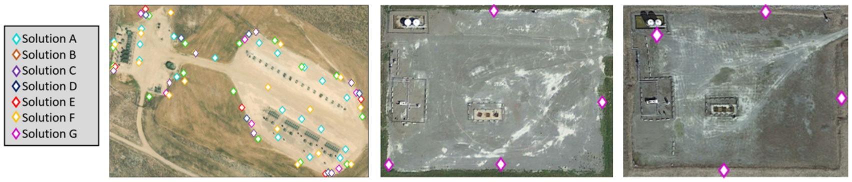

Figure S-2: The aerial images, from left to right, show sites 1, 2, and 3 with areas of 9, 2.5, and 2.5 acres, respectively. Site 1 is a production site in the Upper Green River basin and sites 2 and 3 are production sites in the Marcellus basin. Images from GoogleEarth.

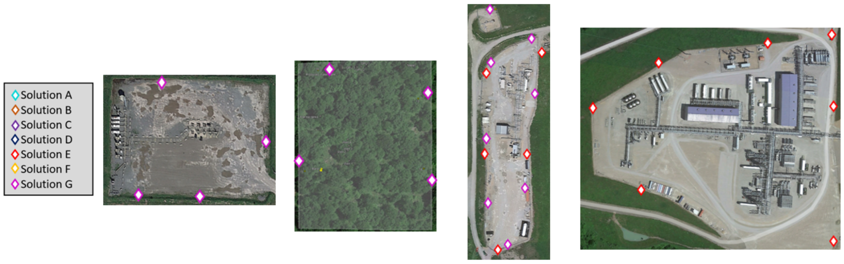

Figure S-3: Satellite images of sites 4, 5, 6, and 7 with areas of 2.5, 2.5, 3.5 and 19 acres, respectively. Sites 4 and 5 are production sites in the Marcellus basin, and sites 6 and 7 are gathering stations in the Utica basin. Images from GoogleEarth.

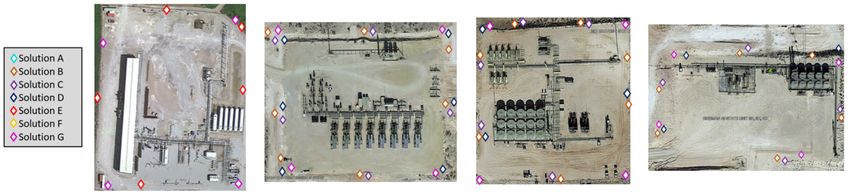

Figure S-4: The aerial images, from left to right, show sites 8, 9, 10, and 11 with areas of 5.5, 6.5, 2.5 and 2 acres, respectively. Sites 8 and 9 are gathering stations and sites 10 and 11 are production sites. Sites 9 – 11 are in the Permian basin and site 8 is in the Utica basin. Images from GoogleEarth.

S-2.2 Challenge Release Equipment

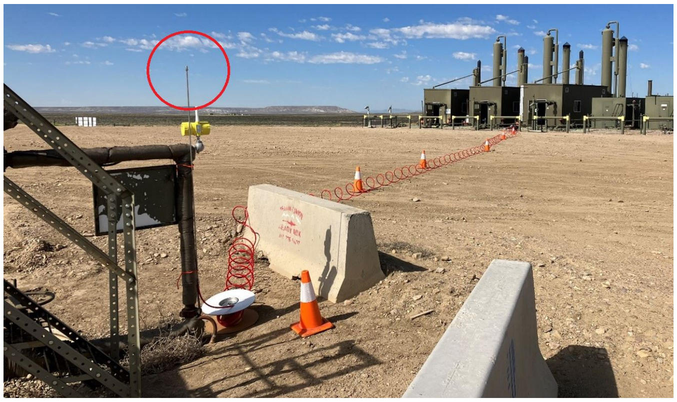

Figure S-5: A challenge release emission point during the campaign located adjacent to a wellhead and supplied gas from the challenge release rig located across the production pad near dehydration and separation equipment.

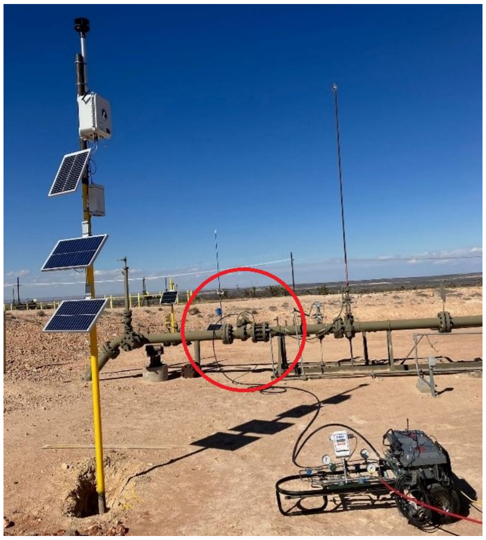

Figure S-6: Mobile release rig with supply inlet connected to the gas line at an oil and gas site during the field campaign.

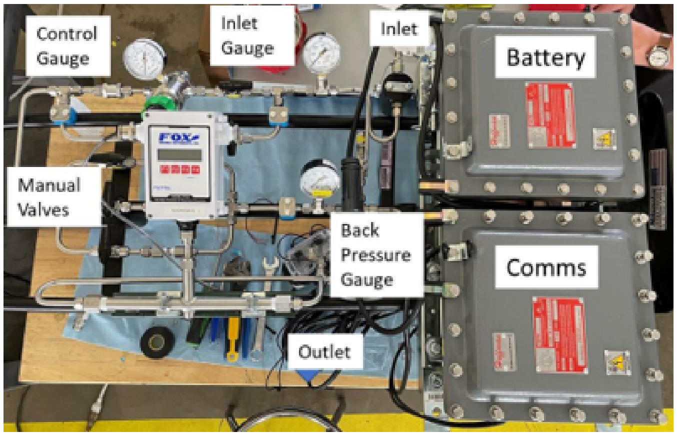

Figure S-7: Release rig constructed by Colorado State University.

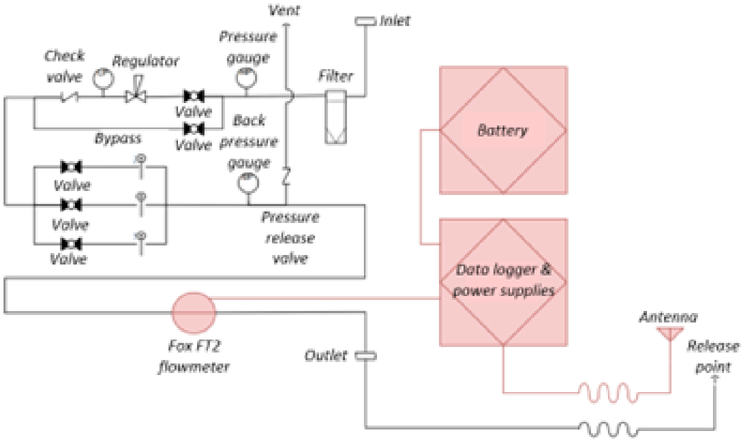

Figure S-8: Release rig flow diagram.

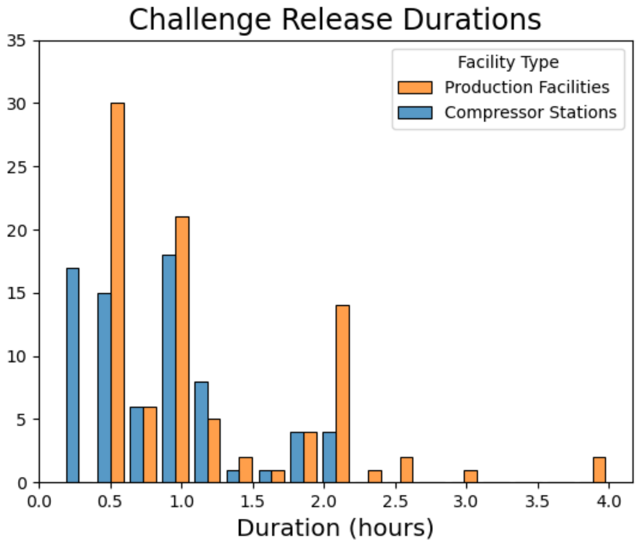

Figure S-9: Challenge release durations at production facilities and compressor stations during the field campaign.

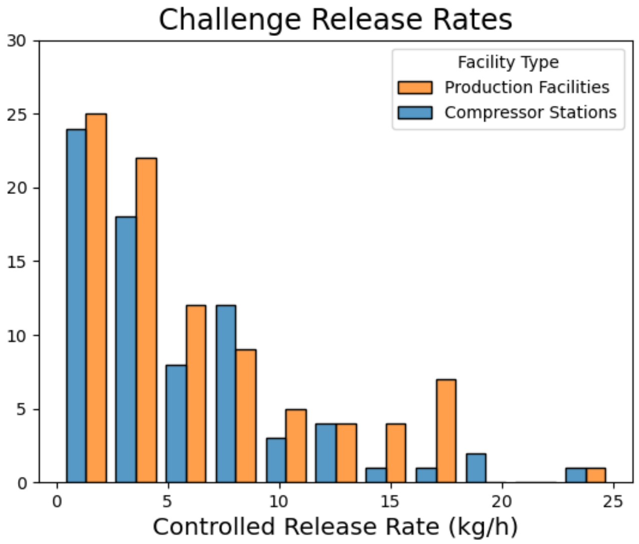

Figure S-10: Challenge release rates at production facilities and compressor stations during the field campaign.

S-2.3 Challenge Release Detection Classification

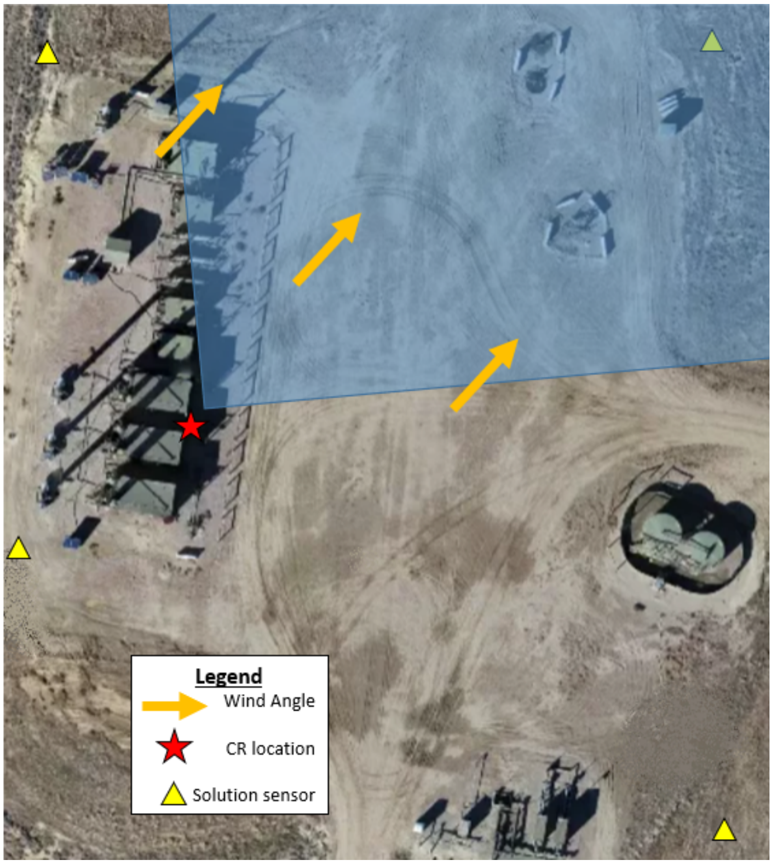

Figure S-11: Image shows an example of a challenge release location, wind angle, and area, in transparent blue, which is considered as a sensor being downwind of the challenge release location.

S-3 Results and Discussion

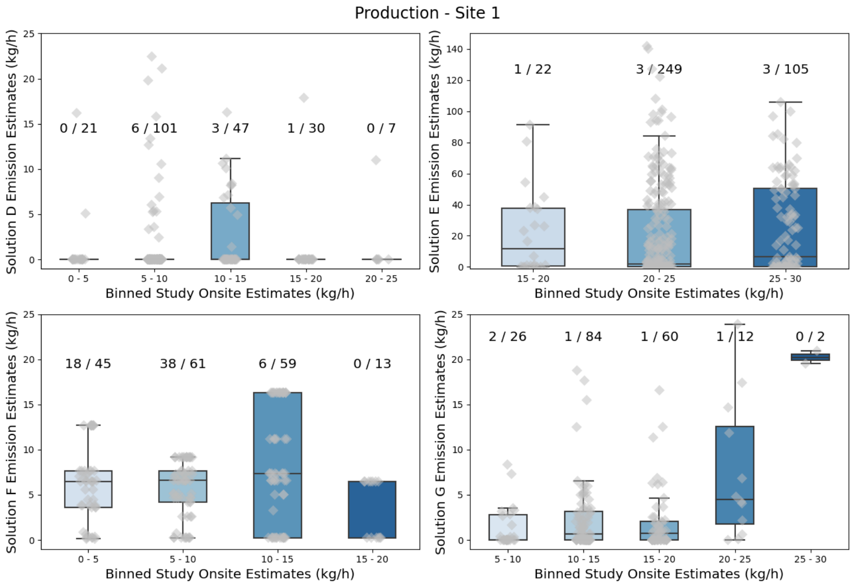

Figure S-12: Plots for Site 1 show the ratio of how many estimates were within the binned ChR rates over the total number of estimates that could have been made within the binned ChR rates. This number of possible estimates is slightly different for each solution at each site, as they provide estimates in different time intervals. Note that estimates of 0 kg/h are shown in the box and scatter plots, but not counted in the ratios.

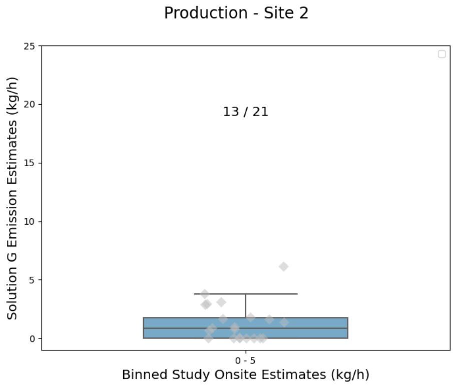

Figure S-13: Plots for Site 2 show the ratio of how many estimates were within the binned SOEs over the total number of estimates that could have been made within the binned SOEs. This number of possible estimates is slightly different for each solution at each site, as they provide estimates in different time intervals. Note that estimates of 0 kg/hr are shown in the box and scatter plots, but not counted in the ratios.

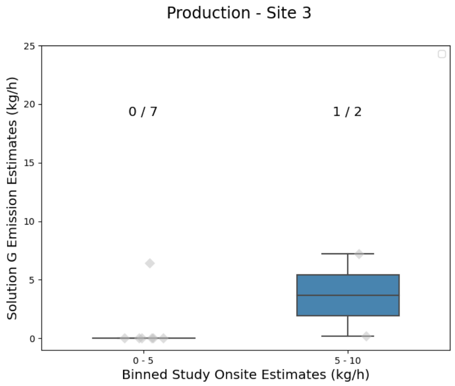

Figure S-14: Plots for Site 3 show the ratio of how many estimates were within the binned SOEs rates over the total number of estimates that could have been made within the binned SOEs rates. This number of possible estimates is slightly different for each solution at each site, as they provide estimates in different time intervals. Note that estimates of 0 kg/hr are shown in the box and scatter plots, but not counted in the ratios.

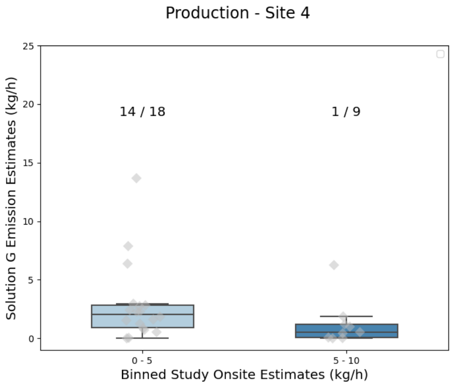

Figure S-15: Plots for Site 4 show the ratio of how many estimates were within the binned SOEs over the total number of estimates that could have been made within the binned SOEs. This number of possible estimates is slightly different for each solution at each site, as they provide estimates in different time intervals. Note that estimates of 0 kg/hr are shown in the box and scatter plots, but not counted in the ratios.

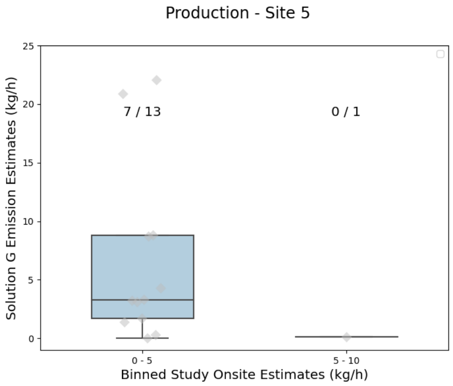

Figure S-16: Plots for Site 5 show the ratio of how many estimates were within the binned SOEs over the total number of estimates that could have been made within the binned SOEs. This number of possible estimates is slightly different for each solution at each site, as they provide estimates in different time intervals. Note that estimates of 0 kg/hr are shown in the box and scatter plots, but not counted in the ratios.

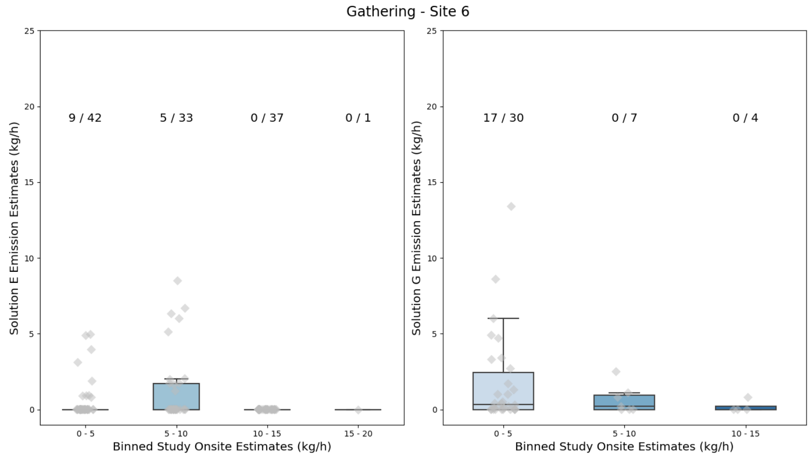

Figure S-17: Plots for Site 6 show the ratio of how many estimates were within the binned SOEs over the total number of estimates that could have been made within the binned SOEs. This number of possible estimates is slightly different for each solution at each site, as they provide estimates in different time intervals. Note that estimates of 0 kg/hr are shown in the box and scatter plots, but not counted in the ratios.

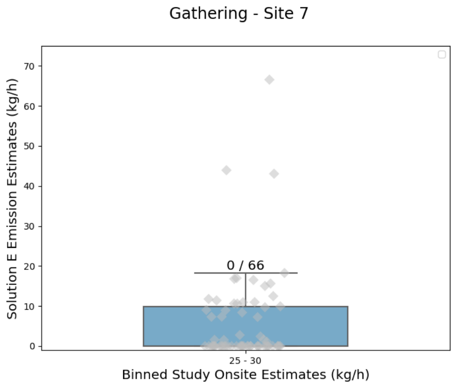

Figure S-18: Plots for Site 7 show the ratio of how many estimates were within the binned SOEs over the total number of estimates that could have been made within the binned SOEs. This number of possible estimates is slightly different for each solution at each site, as they provide estimates in different time intervals. Note that estimates of 0 kg/hr are shown in the box and scatter plots, but not counted in the ratios.

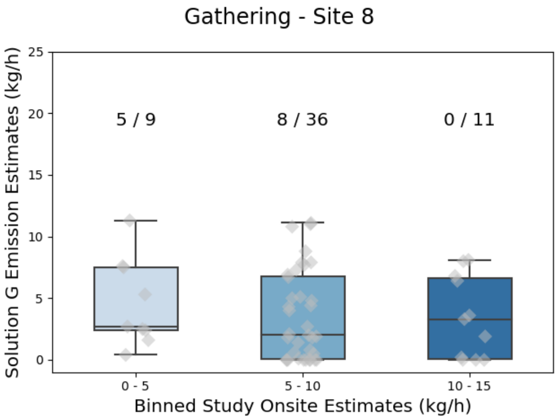

Figure S-19: Plots for Site 8 show the ratio of how many estimates were within the binned SOEs over the total number of estimates that could have been made within the binned SOEs. This number of possible estimates is slightly different for each solution at each site, as they provide estimates in different time intervals. Note that estimates of 0 kg/hr are shown in the box and scatter plots, but not counted in the ratios.

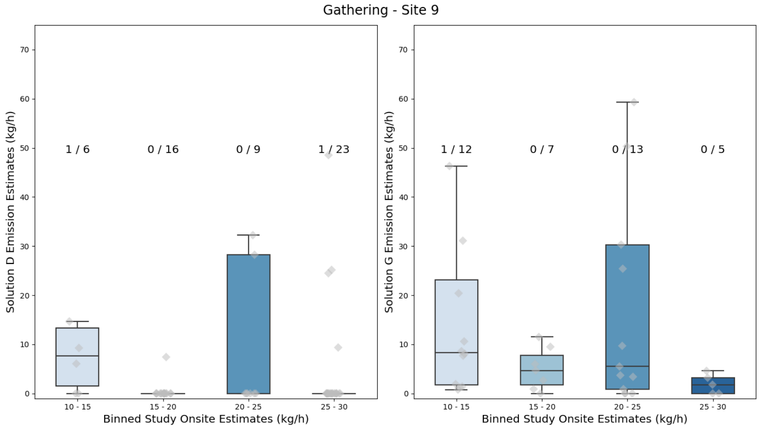

Figure S-20: Plots for Site 9 show the ratio of how many estimates were within the binned SOEs over the total number of estimates that could have been made within the binned SOEs. This number of possible estimates is slightly different for each solution at each site, as they provide estimates in different time intervals. Note that estimates of 0 kg/hr are shown in the box and scatter plots, but not counted in the ratios.

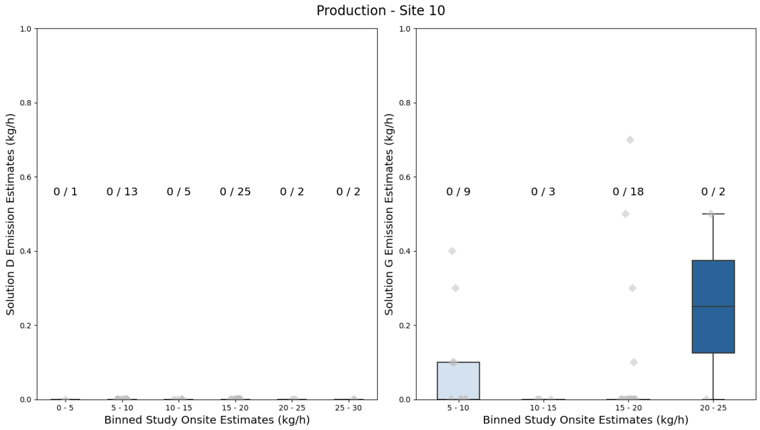

Figure S-21: Plots for Site 10 show the ratio of how many estimates were within the binned SOEs over the total number of estimates that could have been made within the binned SOEs. This number of possible estimates is slightly different for each solution at each site, as they provide estimates in different time intervals. Note that estimates of 0 kg/hr are shown in the box and scatter plots, but not counted in the ratios.

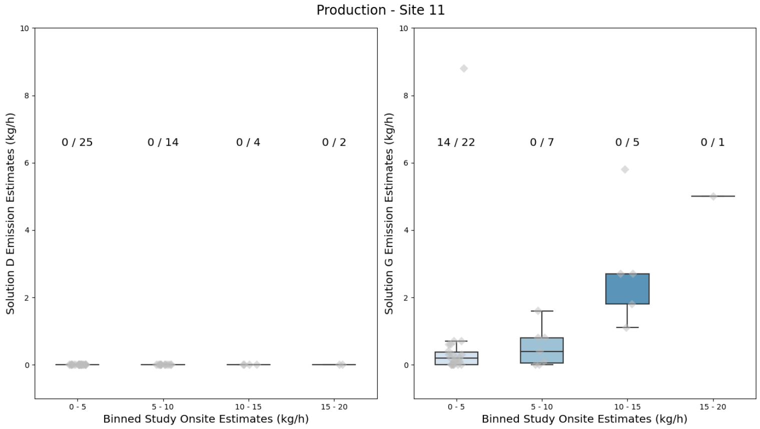

Figure S-22: Plots for Site 11 show the ratio of how many estimates were within the binned SOEs over the total number of estimates that could have been made within the binned SOEs. This number of possible estimates is slightly different for each solution at each site, as they provide estimates in different time intervals. Note that estimates of 0 kg/hr are shown in the box and scatter plots, but not counted in the ratios.

S-3.1 Probability of Detection

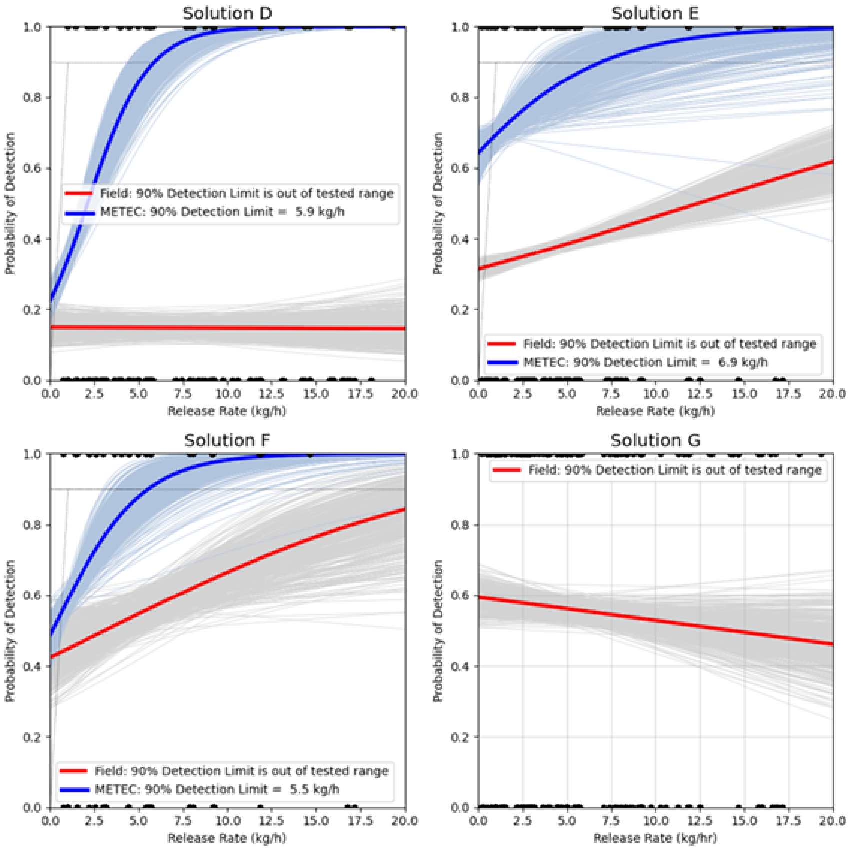

Figure S-23: Probability of detection as a logistic regression curve function of site rate estimates during challenge releases for all solutions during all deployments paired with their estimates made during METEC testing. The definition of detection, or a TP reading, for the field campaign includes any estimate above 0 kg/hr for solutions D, E, and G. As solution F does not have any 0 kg/hr estimates, the definition of detection is any estimate above 2.23 kg/hr, the BL site rate estimate, see *Methods*. Solution G did not participate in METEC testing.

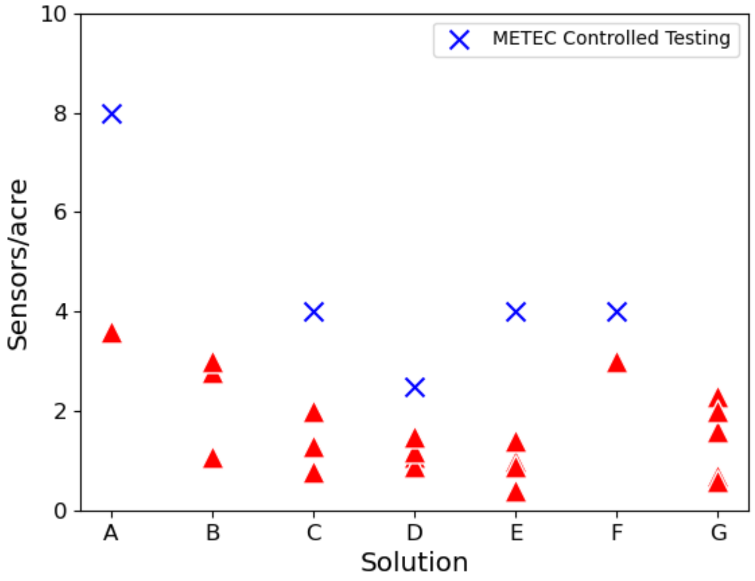

Figure S-24: Number of sensors per area by solution for METEC controlled releases and the field campaign's challenge releases. For the field campaign an average is taken across all sites.

S-3.2 Mixing Ratio Results

Table S-2: Distances between point sensors and challenge releases (ChRs).

Sensor distances from ChRs

Site	*Minimum (m)*	*Maximum (m)*	*Average (m)*
Site 1	17	350	184
Site 2	39	110	75
Site 3	46	100	73
Site 4	44	111	78
Site 5	24	82	53
Site 6	27	132	78
Site 7	37	350	194
Site 8	60	145	103
Site 9	43	152	98
Site 10	38	102	70
Site 11	22	92	57

S-3.3 Site Rate Quantification Results

Table S-3: Baseline (BL) emission estimates (kg/h) at each facility by each solution. Significant variability exists between baseline emissions assessed by solutions deployed at the same facility.

Site Baselines

Site	*Site Type*	*(D)*	*(E)*	*(F)*	*(G)*
Site 1	Production	3.4	19.1	2.2	7.8
Site 2	Production	-	-	-	0.3
Site 3	Production	-	-	-	0.5
Site 4	Production	-	-	-	1.4
Site 5	Production	-	-	-	0.1
Site 6	Compressor	-	0.9	-	0.3
Site 7	Gas plant	-	26.8	-	
Site 8	Compressor	-	70.6	-	4.2
Site 9	Compressor	12.5	-	-	9.1
Site 10	Production	0.3	-	-	0.2
Site 11	Production	0.0	-	-	0.7

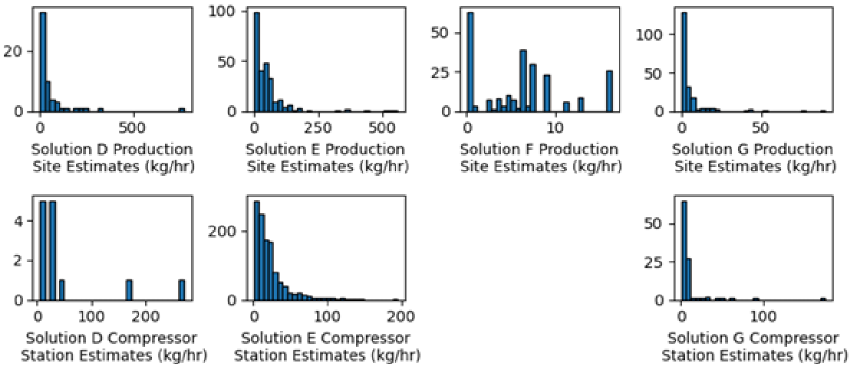

Figure S-25: Histograms show the distribution of site rate estimates from the whole field campaign for each solution with the top row showing production sites and the lower row showing compressor stations.

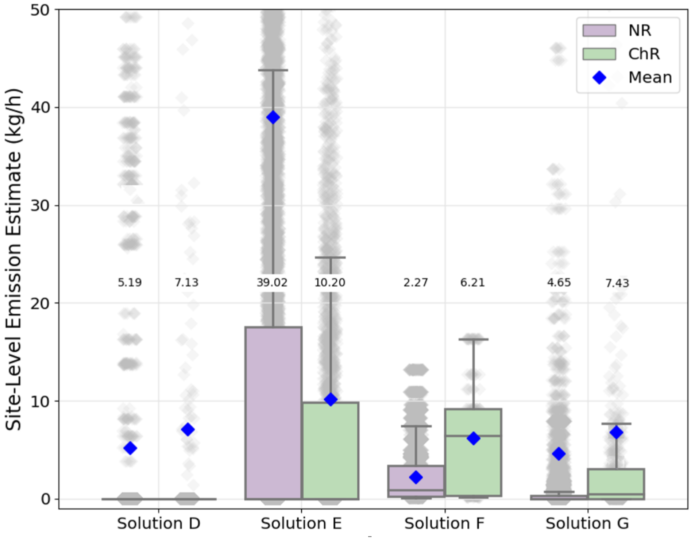

Figure S-26: Averages of site-level emission estimates when ChRs were occurring and when no releases were occuring.
